# Rectal Atresia and Congenital Hypothyroidism: An Association or Coincidence?

**DOI:** 10.1055/s-0037-1612610

**Published:** 2018-01-10

**Authors:** Feride Mehmetoğlu

**Affiliations:** 1 Department of Pediatric Surgery, Dortcelik Children's Hospital, Bursa, Turkey

**Keywords:** rectal atresia, congenital hypothyroidism, posterior sagittal approach

## Abstract

Rectal atresia is a rare anorectal malformation, and its association with other anomalies is even more rare. This study presents a unique case of co-twin in which the surviving newborn male underwent surgery due to rectal atresia. Newborn screening tests identified congenital hypothyroidism. The surgical treatment consisted of three stages and thyroid hormones were replaced.

## Introduction

Isolated rectal atresia is a rare condition with a reported incidence of 1% of all anorectal malformations. 1 2 The exact pathogenesis of rectal atresia is not known, but developmental, embryological, thickened Houston's valves, genetic, infective and environmental theories have been postulated. 1 Most authors believe it to be an acquired lesion due to the lack of associated congenital anomalies. 3

A broad search of the English literature was performed to screen for relevant papers that examined other anomalies associated with rectal atresia, including Down's syndrome, congenital cardiac and renal diseases, tracheoesophageal fistula, choanal atresia, skeletal anomalies, midgut malrotation, presacral masses, pouch colon anomalies, vaginal atresia and vesical, urethral, double urethral, vestibular and labial fistulas of the rectum. 1 2 3 4 5 6 7 8 9 10 11 Unlike rectal atresia, hypothyroidism exhibits a high rate of associated anomalies. 12 13 14 15 The association of rectal atresia with congenital hypothyroidism has not been reported to date in either rectal atresia or congenital hypothyroidism studies in the literature. 1 2 3 4 5 6 7 8 9 10 11 12 13 14 15

In this study, a unique case of rectal atresia that presented as congenital hypothyroidism with isolated rectal atresia is reported in a newborn. The patient was treated successfully by a three stage posterior sagittal approach. This case is presented to demonstrate an uncommon and important therapeutic approach.

## Case Report

**Fig. 1:**
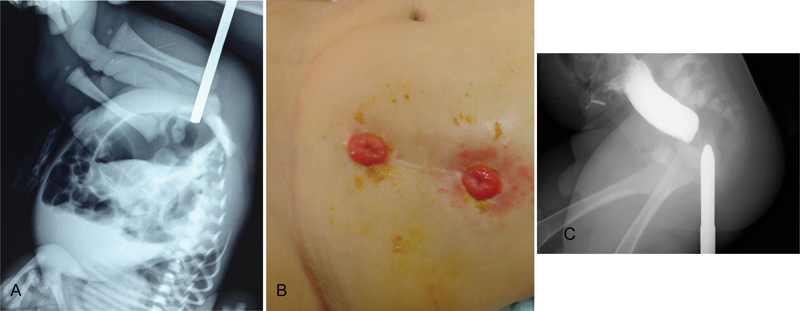
( **A** ) Invertogram showing that the dilator contacted the air column in the distal intestine and the air-fluid levels at 28 hours of age. ( **B** ) Diverting sigmoid colostomy when the patient was 8 months old. ( **C** ) Distal cologram showing the rectal atresia without any fistula with an ∼1.5 cm atretic gap.

A 1-day-old male neonate was admitted due to failure to pass meconium and abdominal distention. At that time, prenatal ultrasonic investigations and the family history were reported as normal. During follow-up, the mother was revealed to have had a high-risk pregnancy. A prenatal ultrasonic investigation at 10 weeks of gestation revealed fetal death of one of twin fetuses. A decision was then made to closely monitor the pregnancy to preserve the remaining fetus. After that, pregnancy went well and no pathologic futures were reported in the surviving baby' ultrasounds. The surviving twin was delivered with a birth weight of 2300 g via elective caesarian section to a 23-year-old primigravida mother after a full-term pregnancy. The patient exhibited progressive abdominal distension, vomiting, and failure to pass meconium. A physical examination revealed a normal appearance of the perineum, genitalia and a normally located anal opening; funnel anus was not detected and no signs of fistula were present. After examination of the anal opening with a firm catheter, a blind-ending anal canal was diagnosed. An upside-down invertogram was performed, and a Hegar dilator was passed through the anal opening, which revealed an air column in the distal intestine ( Fig. 1A ).

Plain X-rays, echocardiography, ultrasound examinations of the newborn did not reveal any associated cardiac, spinal, urinary, or skeletal anomalies; the sacrum and sacral ratio were normal and there was no presacral mass on spinal ultrasound. A diverting descending colostomy was performed on the second day of life ( Fig. 1B ). Biopsy was taken for Hirschsprung's disease and result confirm the presence of ganglion cells. Newborn screening tests identified congenital hypothyroidism, which was confirmed by the results of the blood-free thyroxin (T4) and thyroid-stimulating hormone tests. Therefore, on the 12 days of life thyroid hormone replacement was started at a dose of 11.0 μg/kg/day with levothyroxine.

**Fig. 2:**
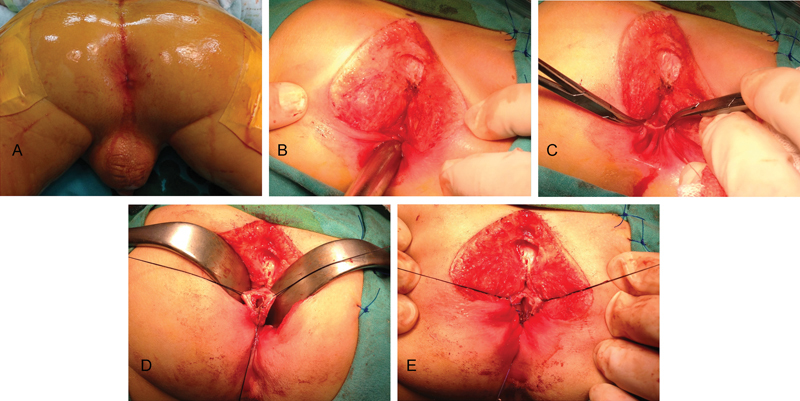
( **A** ) Intraoperative image showing the patient in a prone position with the pelvis elevated for the posterior sagittal approach. The patient exhibited normal perineum, genitalia, and a normally located anal opening. ( **B** ) Exposure of the defect with a Hegar dilator in the distal pouch. ( **C** ) The fibrous tissue separating the two rectal pouches (the incision asymmetry is due to the asymmetric position of the instrument). ( **D** ) The upper pouch of the rectum is held with sutures and opened. ( **E** ) A simple end-to-end circumferential anastomosis with interrupted long-term absorbable sutures.

A definitive operation was performed at the age of 9 months via the posterior sagittal approach. Before the operation, a distal colostogram revealed rectal atresia without any fistula, and the atretic gap length of the rectum was determined to be ∼1.5 cm ( Fig. 1C ). The continuity of the rectum was established with circumferential anastomosis using interrupted absorbable sutures and a posterior sagittal approach ( Fig. 2A – E ). A muscle stimulator was used during the entire procedure, and good muscle contractions were observed equally on both sides ( Video 1 ). Closure of the colostomy was performed after completion of a dilatation program at the age of 1 year. Thyroid hormone replacement continued throughout the follow-up period. The patient is currently well with a follow-up period of 4.5 years and his rectal examination is normal. There is no fecal incontinence or soiling, but he suffers from constipation and requires stool softeners for this condition. Bowel function was assessed according to the Rintala bowel function score; it is classified as a good outcome with 15 points. The hypothyroidism follow-up is continued by the pediatric endocrinology clinic.

**Video 1** A muscle stimulator was used during the operation, and good muscle contractions were observed equally on both sides. Online content including video sequences viewable at: www.thieme-connect.com/products/ejournals/html/10-1055-s-0037-1612610-EJPSR-17-0353-v1.mp4 .

## Discussion

Several classifications are used to define anorectal malformations. Rectal atresia is generally categorized as complex and unusual. 16 Rectal atresia has also been referred as a colonic atresia. 17 Moreover, rectal atresia itself is classified in different manners. Sharma and Gupta updated and revised the rectal atresia classification to incorporate the various types observed and provide increased detail. The present case, which consisted of a gap between the proximal and distal ends, is classified as type four according to their publications and is extremely rare. 1

Congenital hypothyroidism is the most common endocrine disorder, affects ∼1:2,000 to 1:4,000 newborns worldwide and exhibits a high prevalence of extrathyroidal malformations. 12 Common symptoms include goiter, poor feeding, constipation, hypothermia bradycardia, and prolonged jaundice. 12 Infants exhibiting congenital hypothyroidism and extrathyroidal congenital malformations were reported with prevalence rates ranging from 8.4% to 28.2%. The majority of these patients exhibited cardiac features, dysmorphic features, neurologic abnormalities, genitourinary malformations, and Down's syndrome. 12 13 14 15 To the best of our knowledge, the association of hypothyroidism and rectal atresia has not been described so far. Although several explanations of the relationship between hypothyroidism and congenital malformations have been proposed hitherto, none is considered adequate. 13 14 15 Therefore, the coexistence of the hypothyroidism and rectal atresia is related to coincidence or association remains questionable.

Distal intestinal atresia is difficult to diagnose prenatally, and this condition is a very rare cause of intestinal obstruction. 18 Management depends on the level of atresia and the presence of associated anomalies. In cases of rectal atresia with a normal anal opening, a delay in diagnosis and potential complications, such as pneumoperitoneum and mortality, may occur. 11 On examination, anus sometimes appears skin lined, narrowed, and funnel shaped. It is known as a “funnel anus” and highly suggestive for rectal atresia and rectal stenosis. 2 19

Preoperative workup includes plain and cross table or upside-down X-rays, echocardiography, and ultrasonography of the urologic system and spine. Presacral mass should not be overlooked. Hamrick et al reported a 29% prevalence of presacral masses in a rectal atresia/stenosis series. 2

Rectal atresia cases require a preliminary diverting colostomy to minimize postoperative complication. 2 Biopsy may be taken at the time of making a colostomy to rule out associated Hirschsprung's disease. 1 A distal cologram is helpful to exclude fistula and confirm the length of the atretic gap. 1 2 Obstructive uropathies are common in these neonates and warrant urgent decompression of the urinary tract as well as a colostomy. 6

The optimal surgical care for patients with rectal atresia begins with appropriate decision making during the critical newborn period. Many operative approaches are used to correct this unique malformation. 2 3 19 20 An alternative option consisting of treatment of rectal atresia with anastomosis using magnets has been reported. 21 However, the selection of the surgical technique depends on the presence of associated anomalies, presacral mass, pouch colon, and fistula. 2 3 7 8 9 11

Posterior sagittal approach is a useful technique for the surgical correction of rectal atresia according to the largest reported series of rectal atresia/stenosis patients. 1 16 A midline posterior sagittal incision exposes the rectal pouch, which is mobilized from the surrounding muscle fibers, and a direct, end-to-end anastomosis between the blind tips of the anus and rectum can be performed. 16 This technique has been successfully used in cases of rectal atresia with a gap between the two pouches. 1 After the colostomy is closed following dilatation, patients with rectal atresia have appropriate bowel control for their age. 2

Postoperative assessment is done with standardized questionnaires by Rintala and Lindahl at the age of 4.5 years. 22 Constipation in anorectal malformations is extremely common, particularly in the lower types with good prognosis for bowel control which include rectal atresia or stenosis. 2 23 However, among the symptoms caused by hypothyroidism, constipation is foremost, but some patients do not experience complete resolution of hypothyroid symptoms when treated with sufficient hormone therapy. 12 The present patient was continent, but he suffered from constipation. Both hypothyroidism and anorectal malformations are causes of congenital constipation. Therefore, the cause of constipation in our patient on long-term follow-up remains unclear and maybe multifactorial.

## Conclusion

Rectal atresia is very rare, mostly nonsyndromic and occasionally associated with other anomalies. The association of congenital hypothyroidism and rectal atresia is unknown and has not been previously reported. This case report presents the first instance of congenital hypothyroidism and rectal atresia in a male neonate, and the subsequent treatments of both conditions are described. End-to-end rectoanal anastomosis via a posterior sagittal approach is a safe and effective technique for the surgical correction of rectal atresia.

## New Insights and the Importance for the Pediatric Surgeon

A unique case of type four rectal atresia with hypothyroidism was treated with meticulous preoperative examination and PSARP method.
